# Hematoporphyrin Monomethyl Ether-Mediated Photodynamic Therapy Selectively Kills Sarcomas by Inducing Apoptosis

**DOI:** 10.1371/journal.pone.0077727

**Published:** 2013-10-30

**Authors:** Hui Zeng, Mengxiong Sun, Chenghao Zhou, Fei Yin, Zhuoying Wang, Yingqi Hua, Zhengdong Cai

**Affiliations:** 1 Department of Orthopedics, Shanghai First People’s Hospital, Shanghai Jiao Tong University School of Medicine, Shanghai, China; 2 Department of Orthopedics, Shanghai Tenth People’s Hospital, Tongji University School of Medicine, Shanghai, China; 3 The Advanced Institute of Translational Medicine, Tongji University, Shanghai, China; Roswell Park Cancer Institute, United States of America

## Abstract

We investigated the antitumor effect and mechanism of hematoporphyrin monomethyl ether-mediated photodynamic therapy (HMME-PDT) in sarcomas. Intracellular uptake of HMME by osteosarcoma cells (LM8 and K7) was time- and dose-dependent, while this was not observed for myoblast cells (C2C12) and fibroblast cells (NIH/3T3). HMME-PDT markedly inhibited the proliferation of sarcoma cell lines (LM8, MG63, Saos-2, SW1353, TC71, and RD) (P<0.05), and the killing effect was improved with increased HMME concentration and energy intensity. Flow cytometry analysis revealed that LM8, MG63, and Saos-2 cells underwent apoptosis after treatment with HMME-PDT. Additionally, apoptosis was induced after HMME-PDT in a three-dimensional culture of osteosarcoma cells. Hoechst 33342 staining confirmed apoptosis. Cell death caused by PDT was rescued by an irreversible inhibitor (Z-VAD-FMK) of caspase. However, cell viability was not markedly decreased compared with the HMME-PDT group. Expression levels of caspase-1, caspase-3, caspase-6, caspase-9, and poly (ADP-ribose) polymerase (PARP) proteins were markedly up-regulated in the treatment groups and increased with HMME concentration as determined by western blot analysis. In vivo, tumor volume markedly decreased at 7–16 days post-PDT. Hematoxylin and eosin staining revealed widespread necrotic and infiltrative inflammatory cells in the HMME-PDT group. Immunohistochemistry analysis also showed that caspase-1, caspase-3, caspase-6, caspase-9, and PARP proteins were significantly increased in the HMME-PDT group. These results indicate that HMME-PDT has a potent killing effect on osteosarcoma cells in vitro and significantly inhibits tumor growth in vivo, which is associated with the caspase-dependent pathway.

## Introduction

Photodynamic therapy (PDT) has been employed to treat a variety of malignant and benign tumors in recent years. Its efficacy as a curative and palliative therapeutic alternative is well documented. Compared with traditional therapies such as surgery, radiotherapy, and chemotherapy, PDT has advantages like minimal invasion, low toxic side effects, and selective killing of tumor tissue without damage to the surrounding normal structures [1]. Photosensitizer (PS) selectively accumulates in tumor tissues, and type I and II photochemical reactions occur under light irradiation of appropriate wavelengths. Singlet reactive oxygen species (ROS) is generated in these reactions, which lead to photo-damage of tumor cells and have cancer-destroying effects [2]. The anticancer mechanism includes direct induction of apoptosis and necrosis, thereby interrupting tumor blood supply and stimulating an immune response [3]. Currently, PS is widely used for treating malignant tumors such as skin cancer, head and neck cancer, lung cancer, and breast carcinoma [4]–[5].

Osteosarcoma (OS) is the most common primary malignant bone tumor in children and young adults. OS leads to poor limb function and shows high local recurrence and metastasis rates. The conventional treatment for bone and soft tissue sarcoma includes surgical resection, chemotherapy, and radiotherapy, which may lead to severely impaired limb function and a high risk of local recurrence [6]. As a novel treatment modality for bone tumors, PDT is used for patients with advanced stage disease or a large tumor that cannot be ablated by surgery, or those who wish to regain excellent limb function after wide tumor resection [7]. In recent years, surgery combined with PDT has been shown to reduce local recurrence rates, prolong patient median survival time, and maintain favorable limb function [8]. For treating metastatic bone tumors, particularly metastatic spinal tumors, PDT also plays a crucial role in reducing the risk of tumor recurrence, preserving the structural stability of the spine, and improving spinal function [9].

**Figure 1:**
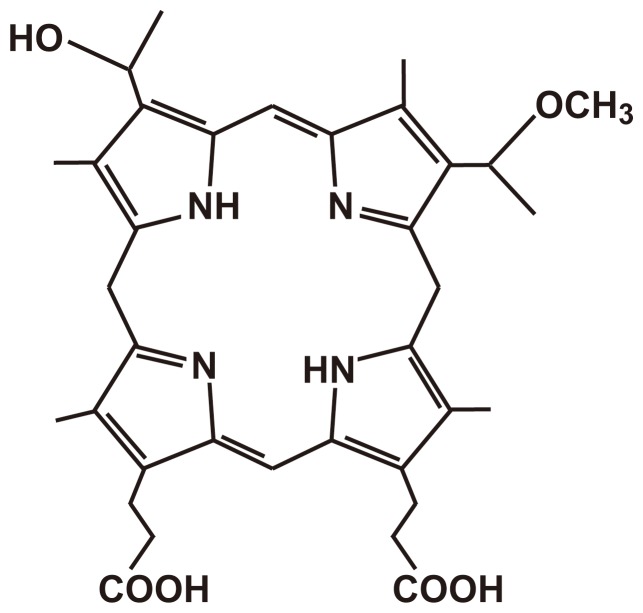
Chemical structure of HMME.

PS is thought to be a critical factor in PDT. Porphyrin has been approved for use in clinical therapy by the US Food and Drug Administration. Hematoporphyrin monomethyl ether (HMME), as a novel second-generation porphyrin-related PS, is a promising PS for PDT. HMME consists of 2 monomer porphyrins, 3-(1-methyloxyethyl)-8-(1-hydroxyethyl) deuteroporphyrin IX and 8-(1-methyloxyethyl)-3-(1-hydroxyethyl) deuteroporphyrin IX (Fig. 1) [10], [11]. Compared with the first-generation PS, HMME has a strong photo-damage effect, shows high ROS generation, more highly selective uptake into tumor tissue, shorter-term skin photosensitization, and low toxicity [11]. Currently, experimental studies and clinical trials have shown that PDT has a significant efficacy on skin diseases as well as benign and malignant tumors [4]. However, HMME-PDT, which has been used in a variety of bone and soft tissue tumors, has not been thoroughly examined.

In this study, we investigated the antitumor effect of HMME-PDT in sarcoma cells and in a mouse sarcoma model. We also explored the mechanisms of cell death of sarcoma in vitro and in vivo. The results of our study provide optimal parameters for HMME-PDT, which will be useful for clinical applications.

## Results

### HMME Selectively Accumulates in Sarcoma Cells

**Figure 2:**
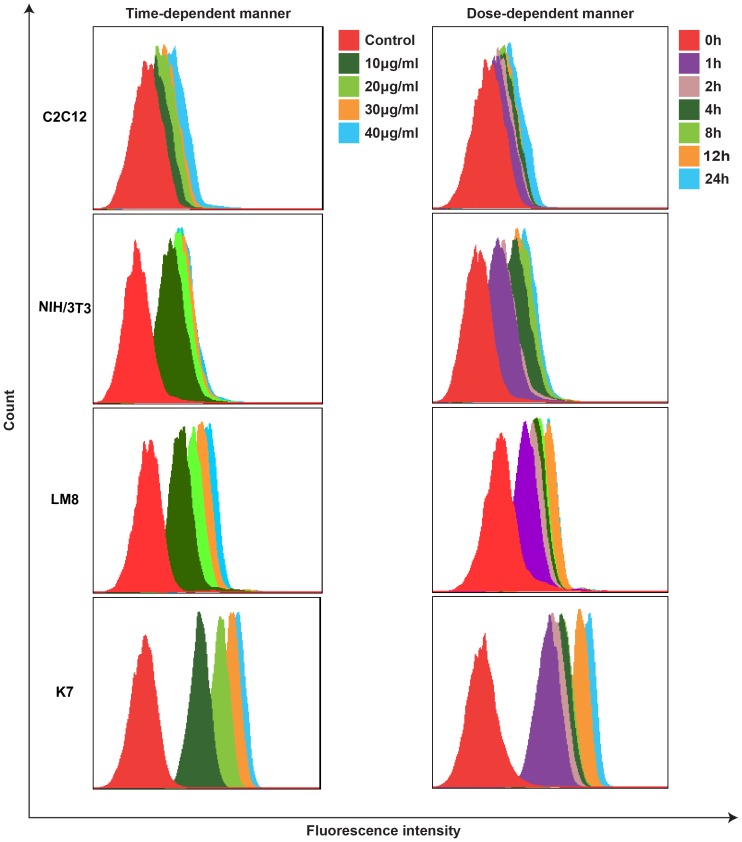
Intracellular uptake of HMME in C2C12, NIH/3T3, LM8, and K7 cells. The intracellular uptake of HMME was analyzed using flow cytometry with a PerCP-Cy5-5-A channel and evaluated based on fluorescence intensity. Fluorescence intensity on sarcoma cells (LM8 and K7)markedly increased with the incubation time and HMME concentrations. However, the fluorescence intensity of myoblast cells (C2C12) and fibroblast cells(NIH/3T3) was not significantly enhanced with increased incubation time or HMME concentration.

Intracellular uptake of HMME was analyzed using FACSCalibur flow cytometry and was evaluated on the basis of the fluorescence intensity recorded. Fluorescence intensity on sarcoma cells markedly increased with the incubation time when the cells were incubated in a medium containing 20 µg/mL HMME. Fluorescence intensity increased with increasing HMME concentrations when cells were incubated with different concentrations of HMME (0, 10, 20, 30, and 40 µg/mL) for 4 h. However, the fluorescence intensity of myoblast cells was not significantly enhanced with increased incubation time or HMME concentration. This result showed that the intracellular uptake of HMME was time- and dose-dependent and that HMME can selectively accumulate in tumor cells, whereas normal cells absorbed less HMME. (Fig. 2).

### HMME-PDT Decreased Sarcoma Cell Viability

**Figure 3:**
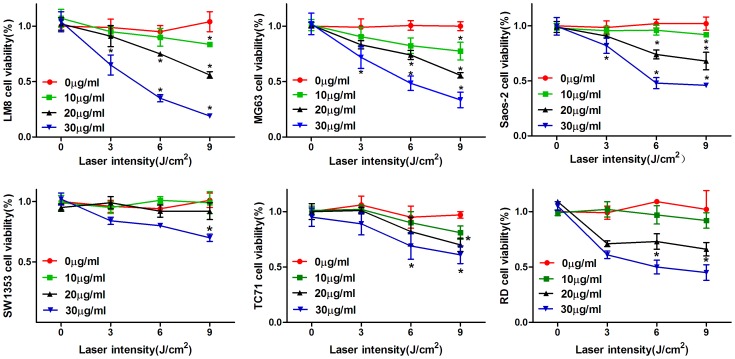
Effects of HMME-PDT on sarcoma cell viability by MTT assay. LM8, MG63, Saos-2, SW1353, TC71 and RD cells were cultured with different concentrations of HMME (10, 20 and 30 µg/mL) for 4 h and then irradiated by laser at different doses (3, 6, 9 J/cm^2^). The control group, PS group and laser group were selected as the control groups. The data were obtained from three independent experiments. *P<0.05 was considered statistically significant by student’s *t* -test, compared with the control group.

The results of the MTT assay showed that HMME-PDT significantly decreased sarcoma cell viability, whereas photo-damage to cells increased with increasing dose of HMME and light intensity. However, laser irradiation or PS alone showed no significant photo-damage to the cells. When the concentration of HMME was 30 µg/mL and the laser irradiation dose was 9 J/cm^2^, the viability of LM8, MG63, Saos-2, SW1353, TC71, and RD cells was 19.0%, 33.4%, 46.0%, 70.0%, 61.0%, and 45.0%, respectively, compared with the control group, which was statistically significant (P<0.05; Fig. 3).

### HMME-PDT Induces Apoptosis of OS Cells in 2D and 3D Culture Environments

**Figure 4:**
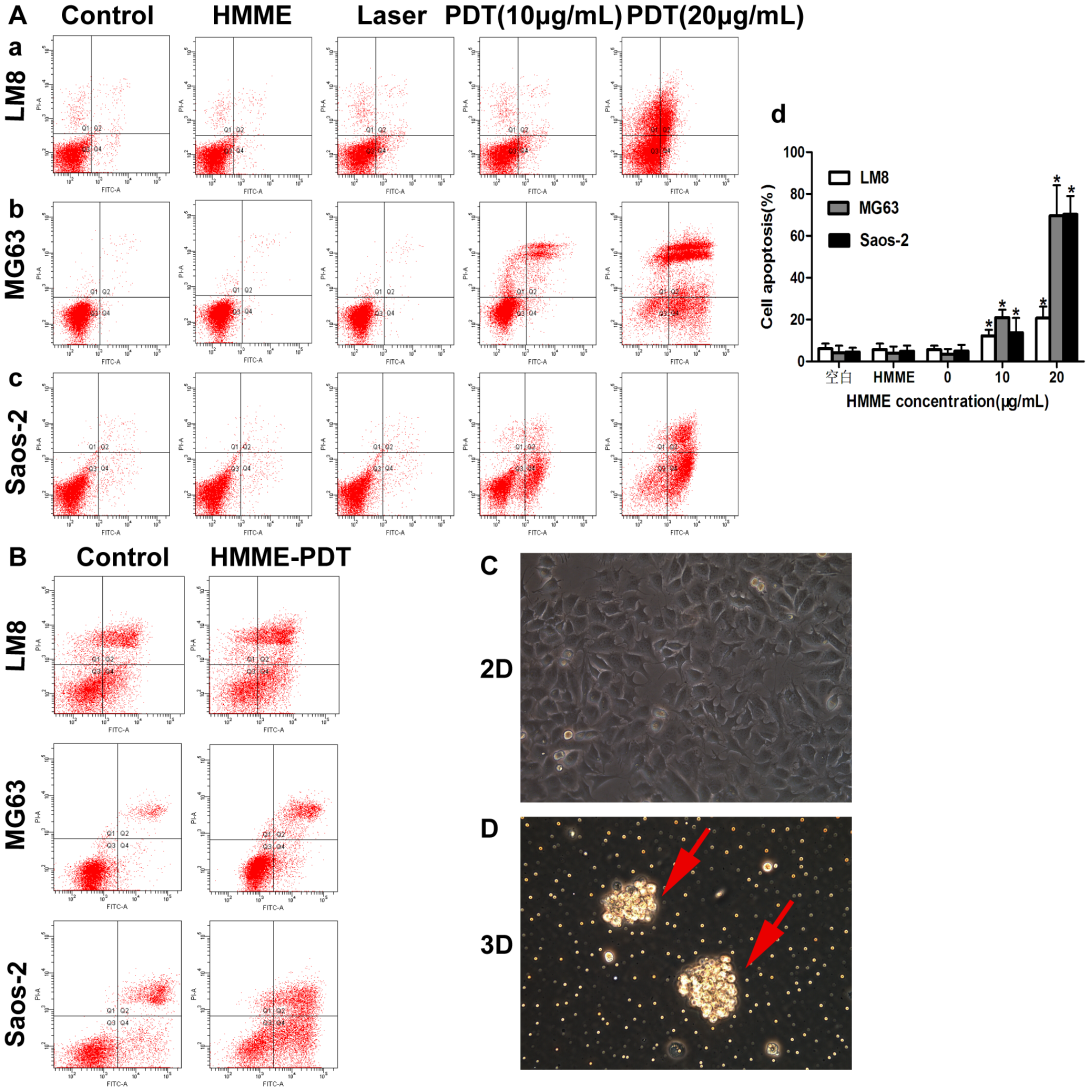
HMME-PDT induces apoptosis of osteosarcoma cells in 2D and 3D culture environments. OS Cells were cultured in 35^2^ petri dish with or without Matrigel and Cells Apoptosis was detected by AnnexinV-FITC/PI staining after HMME-PDT. **A.**HMME-PDT induces apoptosis of OS cells in 2D culture environments. (**a**) Effect of HMME-PDT on apoptosis in LM8 cells. (**b**) Effect of HMME-PDT on apoptosis in MG63 cells. (**c**) Effect of HMME-PDT on apoptosis in Saos2 cells. (**d**) Effect of HMME-PDT on apoptosis of OS cells was analyzed using the CellQuest Software. **B.** HMME-PDT induces apoptosis of OS cells in 3D culture environments. **C.** OS cells were cultured in 2D culture environments. **D.** OS cells were cultured in 3D culture environments. Data was represented with mean±SD from three independent experiments. *P<0.05 was considered statistically significant by student’s *t* -test, compared with the control group.

**Figure 5:**
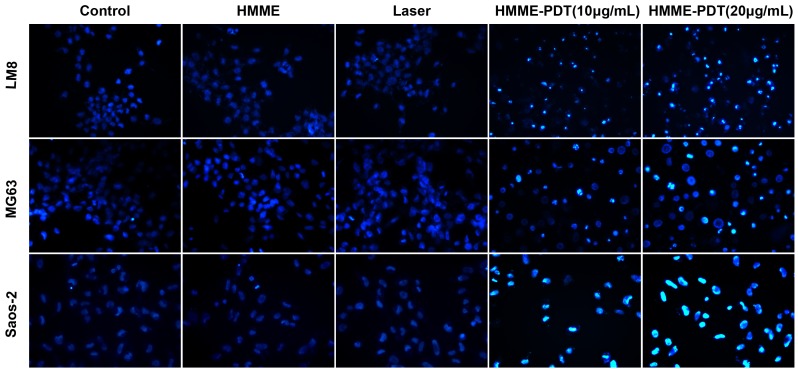
Evaluation of the morphological changes of LM8, MG63 MG63 and Saos-2 cells after HMME-PDT (Hoechst33342 staining, ×400). Changes in apoptosis (nuclear pyknosis and chromatin condensation) were observed under a fluorescence microscope. Apoptotic cells showed bright blue fluorescence and the viable cells showed weak blue fluorescence.

Annexin V-fluorescein isothiocyanate/propidium iodide (FITC/PI) staining was conducted using flow cytometry at 24 h after HMME-PDT. The apoptosis rate of LM8 cells increased by 12.3% and 20.7% in the HMME-PDT group on treatment with 10 and 20 µg/mL, respectively. The total apoptosis rate of MG63 cells significantly increased by 21.0% and 69.6% in the HMME-PDT group on treatment with 10 and 20 µg/mL, respectively. Additionally, the apoptosis rate of Saos-2 cells increased by 13.8% and 70.3% in the HMME-PDT group when treated with 10 and 20 µg/mL, respectively. The percentage of necrotic cells increased by 4.6%, 7.1%, and 1.1% following irradiation at 9 J/cm^2^ (20 µg/mL), respectively. These results show that the apoptosis rate of LM8, MG63, and Saos-2 cells were markedly increased in groups treated with HMME-PDT (10 and 20 µg/mL) compared with the control groups (P<0.05; Fig. 4A). In order to simulate the microenvironment of tumor cells in vivo, three-dimensional culture (3D) was used to investigate the killing mechanism of HMME-PDT. Compared with the control groups, the apoptosis rate of LM8, MG63, and Saos-2 cells increased by 39.5%, 41.2%, and 32.4%, respectively in the HMME-PDT group (Fig. 4B). Changes in apoptosis (nuclei pyknosis and chromatin condensation) upon Hoechst 33342 staining were observed under a fluorescence microscope. These results showed that a larger number of apoptotic cells were observed in the HMME-PDT (10 and 20 µg/mL) groups, and the number increased with increasing concentration of HMME and power intensity. In contrast, few apoptotic cells were detected in the control group and low HMME and laser groups (Fig. 5). Our results indicate that apoptosis is the major type of cell death that occurs after HMME-PDT.

### Caspase-dependent Apoptosis and Necrosis Inhibition

**Figure 6:**
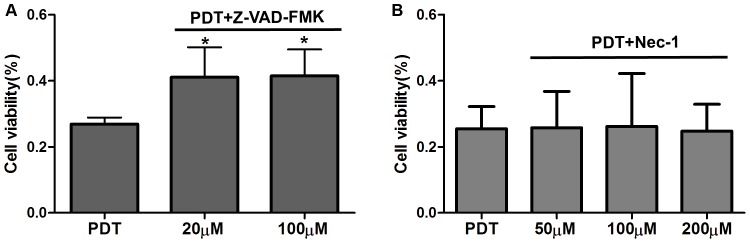
Caspase-dependent apoptosis and necrosis inhibition. Z-VAD-FMK (20 µM and 100 µM) and Nec-1(50 µM, 100 µM and 200 µM) plus HMME (20 µg/mL) were added to cells 4 h before laser irradiation. **A.** Effects of caspase inhibitor on HMME-PDT mediated cell death. **B.** Effects of necrosis inhibitor on HMME-PDT mediated cell death. **P<0.05 was considered statistically significant by Student’s *t*-test and one-way analysis of variance. compared with the control group.

An irreversible inhibitor of caspase (Z-VAD-FMK) and necrosis (Nec-1) was used to determine the form of HMME-PDT-mediated cell death in OS cells. Our results showed that cell viability in the PDT plus Z-VAD-FMK in the 100 µM group and the PDT plus Z-VAD-FMK in the 20 µM group were markedly increased by 14.6% and 14.2%, respectively, compared with the HMME-PDT group. However, addition of Nec-1 (50 µM, 100 µM, and 200 µM) could not rescue cell death (Fig. 6). Our data suggest that apoptosis is the main form of HMME-PDT-mediated cell death and is closely correlated with caspase-dependent apoptosis.

### Activation of a Caspase-independent Apoptotic Pathway after HMME-PDT

**Figure 7:**
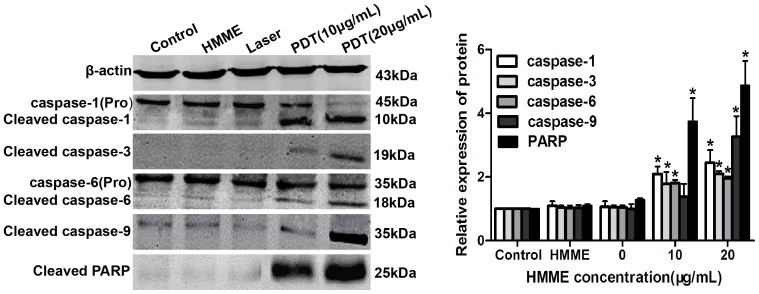
Activation of caspase-independent apoptotic pathway after HMME-PDT. The change of apoptosis related proteins was analyzed using western blotting. The relative expression levels of caspase-1, caspase-3, caspase-6, caspase-9 and PARP proteins were significantly up-regulated in HMME-PDT group. *P<0.05- was considered statistically significant by Student’s *t*-test, compared with the control group.

The caspase family plays significant roles in the execution of tumor cell apoptosis. Compared to the control group, the relative expression of protein was markedly up-regulated in the treatment groups and increased with HMME concentration post-PDT. The cleaved caspase-3 (17 kDa) and caspase-9 (35 kDa) were markedly increased in the HMME-PDT group by 2.10- and 3.27-fold compared to the control group at HMME doses of 20 µg/mL. Procaspase-1 and Procaspase-6 were found to be cleaved, and the cleaved caspase-1 and caspase-6 at 20 µg/mL HMME were 2.45- and 1.96-fold higher than those observed in the control group. The relative expression of poly (ADP-ribose) polymerase (PARP), a nuclear repair enzyme, was also significantly increased with increasing incubation doses of HMME (Fig. 7). These results further suggest that the caspase pathway plays an important role in the initiation of the apoptosis cascade after HMME-PDT in OS cells.

### Tumor Growth was Significantly Inhibited Post-PDT in vivo

**Figure 8:**
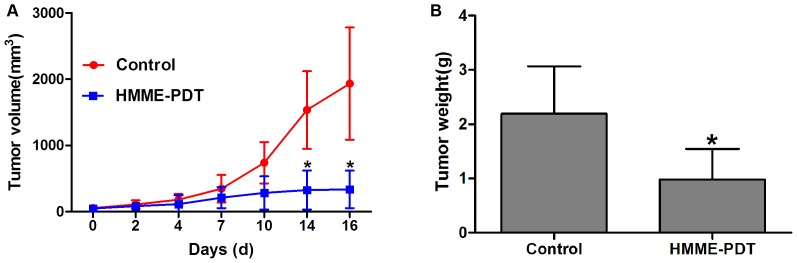
Tumor growth was significantly inhibited post-PDT. HMME (10 mg/kg) was injected into mice caudal vein and the tumor site was irradiated using a laser intensity of 120 J/cm^2^ after 4 hours and 15 min was selected as the laser irradiation time. The sizes and the weights of tumors were observed once every 2–4 days. **A.** tumor volume was markedly reduced in the HMME-PDT group at 14 and 16 days. **B.** Tumor weight was markedly decreased in the HMME-PDT group, compared with the control group. (Control, n = 10; HMME-PDT, n = 10). *P<0.05 was considered statistically significant by Student’s *t*-test, compared with the control group.

To further examine the antitumor activity of HMME-PDT in vivo, we treated OS-bearing mice with HMME plus irradiation. The results showed that tumor volume was significantly decreased post-PDT compared with the control group (P<0.05). Particularly, a significant effect was detected at 14–16 days post-PDT (Fig. 8A). Additionally, tumor weight was markedly reduced compared to the control group (P<0.05; (Fig. 8B). These results indicate that HMME-PDT significantly inhibits tumor growth.

### Histological Changes and Immunohistochemistry Detection after HMME-PDT

**Figure 9:**
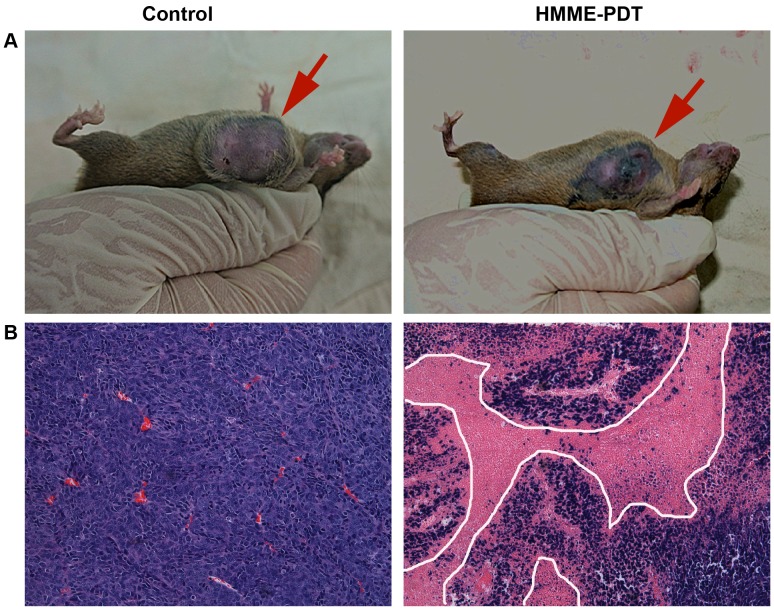
Histological changes of tumor tissue post-PDT by H&E staining (×200). The necrotic areas of tumor dyes red isotropicly undergoing light microscopy. **A.** The tumor surface appeared the widespread necrosis and incrustation region in HMME-PDT group under gross appearance. **B.** The widespread necrotic areas and significant inflammatory cells infiltrative growth were observed in tumor tissues in the HMME-PDT group.

**Figure 10:**
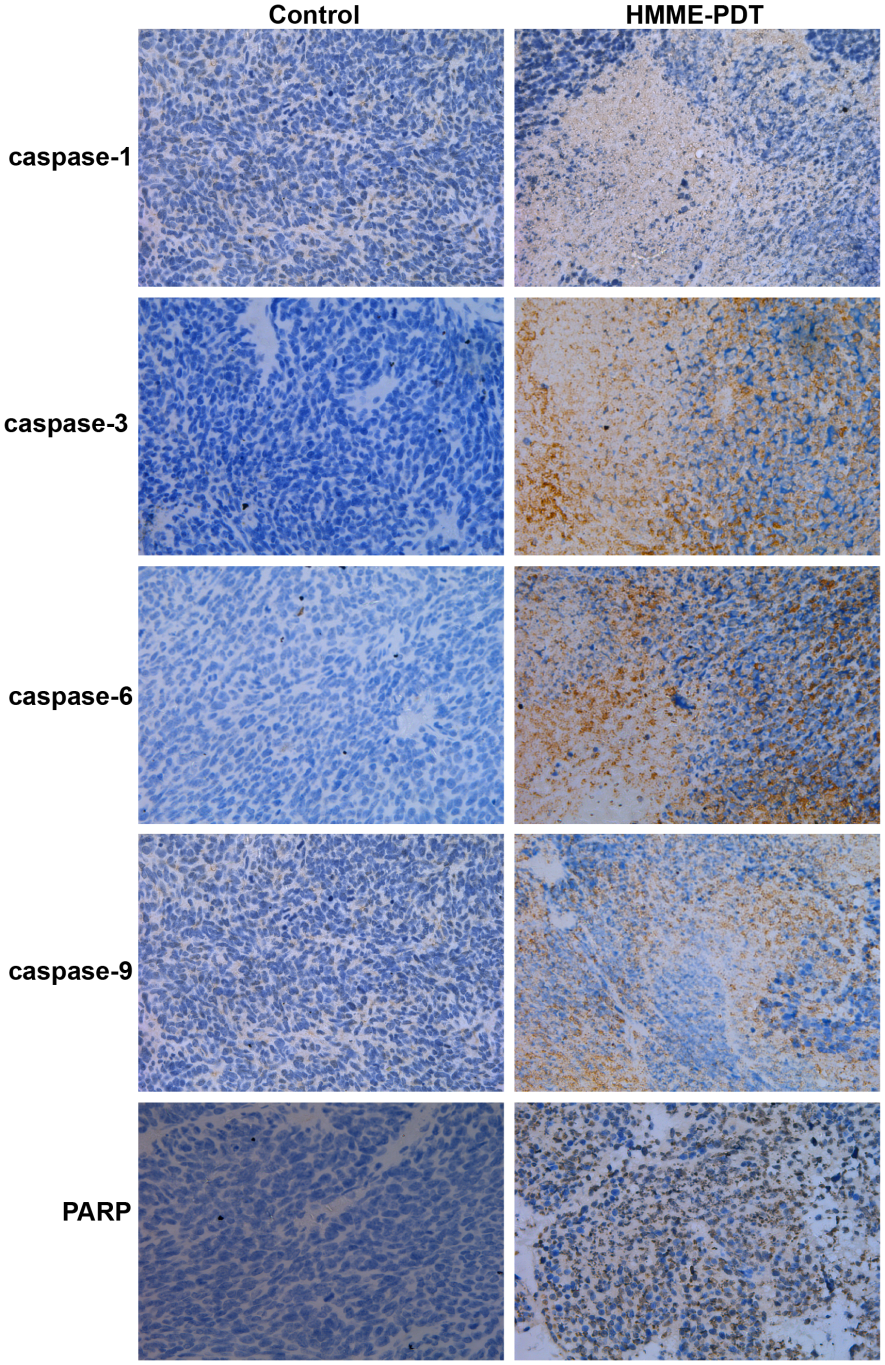
Immunohistochemistry (IHC) detection after HMME-PDT (×200). Tumors were cut and fixed with 10% formalin overnight and specimens were embedded in paraffin and cut into 4-µm-thick sections. Expression of caspase-1, caspase-3, caspase-6, caspase-9, and PARP proteins were evaluated by IHC.

The results of hematoxylin and eosin (HE) staining showed that widespread necrotic areas and significant inflammatory cells infiltrative growth were observed in tumor tissues in the HMME-PDT group. However, there was no appreciable change in the control group (Fig. 9). To future investigate the potential link between the caspase family activation and the antitumor activity of HMME-PDT, immunohistochemistry (IHC) was employed to measure the relative expression of caspase-1, caspase-3, caspase-6, caspase-9, and PARP proteins; the results showed these proteins were primarily expressed in the cytoplasm of tumor tissue. Additionally, the expression of these proteins was significantly increased in the HMME-PDT group compared with the control group (Fig. 10).

## Discussion

PDT, a novel, minimally invasive treatment, has been proved effective for treating head and neck cancer, lung cancer, breast carcinoma, and other cancers [4]–[5]. Additionally, it has marked advantages such as preserving excellent limb function, reducing local recurrence rates, and adjuvant therapy for unresectable tumor in musculoskeletal systems [7], [8], [12]. Compared with surgery, radiotherapy, and chemotherapy, PDT is generally safe on the surrounding normal tissue because PS can selectively accumulate in tumor tissues, whereas accumulation in normal tissues is rarely observed [2]. As a novel, second-generation porphyrin-related PS, HMME has already been used in preclinical trials and experimental studies for PDT of solid tumors and shows promise as an antitumor treatment [12]. Liu demonstrated that HMME-PDT can induce apoptosis of canine breast cancer cells [13]. Zhan reported that HMME-PDT inhibits the growth of glioma and prolongs survival time in vivo [14]. However, the efficacy of HMME-mediated PDT for OS and optimal parameters of treatment have not been reported. In this study, we found that the intracellular uptake of HMME in sarcoma cells was time- and dose-dependent. Fluorescence intensity increased with the incubation time and concentration of HMME. PS can selectively accumulate in tumor cells, but cannot accumulate in normal cells. Additionally, our data suggest that HMME-PDT markedly inhibits sarcoma cell growth, and photodynamic damage of HMME on sarcoma cells increases with the HMME does and light intensity. When the concentration of HMME was 30 µg/mL and the light dose was 9 J/cm^2^, cell viability (LM8, MG63, Saos-2 SW1353, TC71, and RD) reached 19.0%, 33.4%, 46.0%, 70.0%, 61.0%, and 45.0%, respectively (P<0.05). However, laser irradiation or PS alone showed no cytotoxic effect. It is well known that both PS and laser irradiation are indispensable and neither have toxic side effects on cell organization. The data also suggest that it is important to select an appropriate PS concentration, laser irradiation dose, and incubation time in the future clinical treatment of bone tumors.

Apoptosis and necrosis are considered to be the main pathways of PDT-mediated cell death due to direct or indirect damage to tumor cells by singlet ROS [15]. It was previously demonstrated by preclinical research that PDT can induce apoptosis in tumor cells in vitro, whereas cell-killing effects mediated by PDT depend on PS concentrations and laser energy density [16]. To detect the mechanism of PDT-mediated cell death in OS cells, we performed flow cytometry, Hoechst 33342 staining, and treatment with irreversible inhibitor of caspase and necrosis after treatment with HMME-PDT. Some apoptotic cells were detected in the control group, and the apoptosis rate increased by 20.7%, 69.6%, and 70.3% in the HMME-PDT (20 µg/mL) group compared with the control group (P<0.05). However, a significant increase in necrotic cells was not detected in treatment groups. Conventional 2D cell culture lacks the close cellular contact, material metabolism, and endocrine role features of 3D cell culture, which plays an important role in tumor cell proliferation, invasion, and signaling transduction [17], [18]. Two-dimensionally cultured cells grow in the presence of sufficient oxygen and nutrients. Due to hypoxia and endocrine changes, 3D cultured cells are more susceptible to apoptosis than are conventional 2D cultured cells [19]. Three-dimensional culture was first applied to PDT; we found that apoptosis of OS cells could be induced in 2D and 3D cell culture environments and that apoptosis is the main form of cell death by HMME-PDT. Formation of apoptotic bodies and chromatin condensation are remarkable features in apoptosis and can be visualized by the fluorescence of Hoechst 33342 lableing [20]. Chromatin condensation and nuclear pyknosis were observed with Hoechst 33342 in HMME-PDT groups. Additionally, apoptosis may be closely correlated with the caspase pathways.

Although tumor cell death can be induced by PDT, these mechanisms may differ according to various targeting cells, PS, and laser irradiation dose [21], [22]. We found that apoptosis of OS cells may be closely associated with caspase cascade pathways. Therefore, western blotting was employed to determine the correlation between caspase activation and HMME-PDT. A previous study showed that PDT initiates the apoptosis cascade reaction primarily by activating caspase-dependent intrinsic and extrinsic pathways [23]. In the caspase family, caspase-1, caspase-3, caspase-6, and caspase-9 are major cascade activation proteins, and PDT initiates the apoptosis cascade reaction primarily by activating caspase-dependent intrinsic and extrinsic pathways; this cleaves PARP, ultimately leading to apoptosis of tumor cells [24]. First, PDT can activate caspase-8 and caspase-9, and then the active form of caspase-8 and caspase-9 initiates caspase-3 and caspase-6 activation to subsequently activate the remainder of the caspase cascade, which degrades PARP, and finally leads to apoptosis of tumor cells [25], [26]. Liu indicated that the caspase-3/-8 cascade pathway plays a key role in apoptosis of prostate cancer cells induced by PDT [27]. Zhou demonstrated that Casticin enhances TRAIL-induced apoptosis through the up-regulation of expression levels of caspase-1, 3, 6, 9 and PARP proteins [28]. In the present study, the relative expression of caspase family proteins was measured. The results showed that the relative expression of cleavage of caspase-1, caspase-3, caspase-6, caspase-9, and PARP proteins was significantly up-regulated in HMME-PDT groups. IHC also showed that caspase-1, caspase-3, caspase-6, caspase-9, and PARP proteins were mainly expressed in the cytoplasm of tumor tissue. Additionally, expression of these proteins was significantly increased in the HMME-PDT group compared with the control group. This result demonstrates that the antitumor effect is closely correlated with caspase cascade pathways after HMME-PDT. Therefore, our data increase the understanding of the mechanisms involved in the antitumor effect of HMME-mediated PDT on OS. In future studies, we may enhance the killing effect of PDT by specifically inhibiting or activating a pathway.

In vivo, PDT can effectively reduce local recurrence rates and inhibit tumor growth [29]. Recently, PDT was shown to have a significant effect on reducing the local recurrence rate, providing excellent limb function, and prolonging the median survival time of patients with surgical therapy combined with PDT [30]. PDT can be applied to kill residual tumor cells after tumor resection as a new adjuvant therapy [31]. Twenty-six cases of soft tissue sarcoma were treated with surgery combined with PDT. The results showed a low local recurrence rate (7.7%), local recurrence-free rate (88%), and limb function of all patients at 100% of ISOLS criteria [30]. Our data also showed that tumor volume was markedly reduced in the PDT group. Particularly, compared with the control group, a significant effect was observed at 14–16 days post-PDT (P<0.05). Additionally, tumor weight also was markedly reduced compared to the control group (P<0.05). The limited light penetration depth of PDT also affects the ability to treat a tumor because bone and soft tissue tumor are often located in deep areas, where the laser cannot reach. The tumor should not be treated with PDT alone. HE staining showed that widespread necrotic areas and many inflammatory cells capable of infiltrative growth were observed in the superficial areas of tumor tissues in the HMME-PDT group. A wavelength of 635 nm was selected to excite the HMME for the following reasons: First, the absorption peak of HMME is 405, 504, 540, 560, 630 nm while the wavelength of 635 nm can have the best penetration into the tissue. Second, laser irradiation showed no damage to surrounding tissues. However, the tumor continued growing in the deep layers, which is closely correlated with the depth of laser penetration. Typically, bone and soft tumors are located in deeper regions. Because of the extremely limited penetration depth of ordinary light, the efficacy of treatment of bone tumors is severely limited. Therefore, PDT may be an adjuvant treatment after tumor resection, which can preserve normal tissues such as vessels, nerves, and joints and can reduce local recurrence by killing residual tumor cells near the curettage area. Studies have shown that low-dose X-rays and ultrasound can penetrate deeply into tissue and show similar anticancer mechanisms as laser-mediated PDT in vitro and in vivo [32], [33]. Interstitial light delivery technologies are being applied for PDT in head, neck and other cancers [34]. The results of some studies revealed that PDT using interstitial light delivery had a remarkable cytocidal effect and an in vivo and in vitro anti-metastatic and anti-invasive effect [35]. In the future, stronger penetration of the excitation equipment will be studied and applied for treating bone tumors. In conclusion, HMME-PDT can significantly inhibit the viability of OS cells, and the photodynamic damage of HMME on cells was increased with HMME dosage and laser power intensity. Our results also showed that apoptosis is the main form of OS cell death. HMME-PDT may significantly inhibit bone tumor growth. The antitumor effect of HMME-PDT is closely correlated with caspase cascade pathways. With its minimal invasiveness, low-toxic side effects, and highly selective treatment modality, PDT will play an important role in limb salvage treatment and reduction of the local recurrence rate of bone tumors.

## Materials and Methods

### Chemicals

Rabbit monoclonal antibody against caspase-1, caspase-3, caspase-6, caspase-9, PARP, and β-actin and goat or mouse anti-rabbit IgG were obtained from Epitomics, Inc. (Burlingame, CA, USA). An irreversible inhibitor of caspase (Z-VAD-FMK) and necrosis (necrostatin-1, Nec-1), 3(4,5-dimethyl-thiazoyl-2-yl) 2, 5-diphenyltelrazolium bromide, a yellow tetrazole (MTT), and Matrigel were purchased from Sigma-Aldrich (St. Louis, MO, USA). AnnexinV-FITC/PI and Hoechst 33342 were obtained from BD Biosciences (East Rutherford, NJ, USA). HMME was obtained from the Pharmacy Institute of the Second Military Medical University (Shanghai, China). HMME was dissolved in 0.1 M NaOH and the pH was adjusted to 7.0 by adding 0.1 M HCl. Phosphate-buffered saline (PBS) and Dulbecco’s modified Eagle medium (DMEM) containing 10% fetal bovine serum (FBS) at a concentration of 0 µg/mL, 10 µg/mL, and 20 µg/mL was stored in the dark at −20°C. The 630-nm laser equipment was purchased from Guilin Xingda Pharmaceutical Co. (Guangxi, China).

### Cells Culture

A mouse-derived LM8 cell line was obtained from the Chinese Academy of Sciences and human-derived MG63, Saos-2 SW1353, TC71, RD, and mice-derived C2C12, NIH/3T3, K7 cell lines were obtained from the American Type Culture Collection (ATCC; Manassas, VA, USA) and cultured in DMEM medium containing 10% FBS with 100 U/mL penicillin and 100 µg/mL streptomycin. Cells were maintained at 37°C in a humidified atmosphere of 5% CO_2_.

### OS Model

Four-week-old female C3H mice (n = 24; Chinese Academy of Sciences, Shanghai, China) were randomly allocated to the PDT group (12 mice) and control group (12 mice). Mice were housed under standard conditions with a 12-h light-dark cycle and fed plenty of water and food. Single-cell suspensions containing 2×10^6^ LM8 cells in 200 µL of physiological saline were subcutaneously injected into the armpit site under general anesthesia (amobarbital). All animals were kept in a dark room after PDT. All procedures were approved by the animal care and use committee of Shanghai Tongji University. All surgery was performed under sodium pentobarbital anesthesia, and all efforts were made to minimize suffering. Finally, all animals were euthanized by CO_2_ asphyxiation.

### Intracellular Uptake of HMME

LM8 and C2C12 cells were cultured at 35 mm^2^ with 2×10^5^ cells per petri dish and cultured overnight. Next, 2 mL DMEM containing HMME (20 µg/mL) was added to each petri dish and incubated at various time points (0, 1, 2, 4, 8, 12, and 24 h). Cells were incubated with different concentrations of HMME (0, 10, 20, 30, 40 µg/mL) for 4 h. HMME was removed and washed 3 times with PBS. Cells were then centrifuged and resuspended in PBS. The intracellular uptake of HMME was analyzed using FACSCalibur flow cytometry (FCM; Becton Dickinson) with a PerCP-Cy5-5-A channel and evaluated based on fluorescence intensity.

### Cells Viability Assay by MTT Assay

The effects of HMME-PDT on sarcoma cell (LM8, MG63, Saos-2, SW1353, TC71, and RD) viability were evaluated using an MTT assay. Cells were seeded into 96-well plates at a density of 8000/well in DMEM culture medium. After overnight growth, cells were cultured with different concentrations of HMME (10, 20, and 30 µg/mL) for 4 h and then irradiated with a laser at different laser irradiation doses (3, 6, 9 J/cm^2^). Additionally, the blank control group (without PS or irradiation), PS group (PS only), and laser group (irradiation only) were selected as control groups. After 24 h, 20 µL MTT was added to each well and incubated for 4 h at 37°C. The culture medium was removed and 150 µL dimethylsulfoxide (DMSO) was added, then the optical density (OD) was measured at 490 nm using an enzyme-linked immunosorbent assay (ELISA) after 15 min. Cell viability was calculated using the following equation: Cell viability (%) = (OD_490 nm_ of treatment/OD_490 nm_ of blank control)×100%.

### Effect of HMME-PDT on Apoptosis of OS Cells in 2D and 3D Culture Environments

LM-8, MG63, and Saos-2 Cells (2×10^5^/well) were cultured in 35-mm^2^ petri dishes with or without Matrigel overnight. Cells were incubated with different concentrations of HMME (10, 20 µg/mL) for 4 h and then irradiated with a laser at a power intensity of 9 J/cm^2^ for 2 min. After 24 h, cells were washed with cold PBS 3 times, then centrifuged and resuspended in 100 µL of binding buffer in an FCM tube. Next, 5 µL of AnnexinV-FITC and 5 µL of propidium iodide (PI) were added into each FCM tube and incubated in the dark at room temperature for 15 min before analysis. Apoptosis was detected by FCM and analyzed using CellQuest Software. Dual parameter dot plots combining Annexin-V-FITC/PI revealed living cells in the lower-left quadrant (Annexin-V^−/^PI^−^), early apoptotic cells in the lower-right quadrant (Annexin-V^+^/PI^−^), late apoptotic cells in the upper-right quadrant (Annexin-V^−/^PI^+^), and necrotic cells in the upper-left quadrant (Annexin-V^+^/PI^+^). The total apoptosis rate of cells was calculated as the sum of the rates of cells observed in the lower-right quadrant and the upper-right quadrant.

### Hoechst 33342 Staining

Cells (2×10^5^/well) were incubated in a 6-well plate, and the culture medium was removed after HMME-PDT. After washing the cells twice with cold PBS, the cells were stained with Hoechst 33342 for 15 min at 37°C in the dark and then immediately observed to identify LM-8 cell death under a fluorescence microscope (Olympus Inc., Tokyo, Japan).

### Mechanism of PDT-mediated Cell Death by Z-VAD-FMK and Nec-1

To evaluate the mechanism of PDT-mediated cell death after HMME-PDT, an irreversible inhibitor of caspase (Z-VAD-FMK) and necrosis (Nec-1) conjugate PDT was used to detect cell viability. Cells were grown overnight in 96-well plates at a density of 8000 per well. Z-VAD-FMK (100 µM and 20 µM) and Nec-1 (50 µM, 100 µM, and 200 µM) plus HMME (20 µg/mL) were added to cells 4 h before laser irradiation. The HMME-PDT group was used as the control group. After 24 h, cell viability for each experimental group was assayed using the MTT assay.

### Western Blotting Analysis

To further demonstrate the mechanism of caspase-independent apoptosis after HMME-PDT, LM8 cells were harvested and the total protein was extracted with the radioimmuno precipitation assay buffer (50 mM Tris HCl pH 8, 150 mM NaCl, 0.1% SDS, 0. 5% sodium deoxycholate, 1% NP-40) after HMME-PDT. The procedure was performed under ice-cold conditions for 30 min, and then, cells were subjected to shock treatment intermittently. Lysates were centrifuged at 12,000 rpm for 10 min, and supernatants were removed to new Eppendorf tubes and the concentration of protein was determined using the BCA protein assay kit. Proteins (30 µg) were resolved by 8–12% SDS-PAGE and transferred to nitrocellulose (NC) membranes. After blocking with TBST containing 5% skim milk for 1 h, the membranes were incubated with primary antibodies (caspase-1, caspase-3, caspase-6, caspase-9, PARP, and β-actin) overnight at 4°C, followed by incubation with secondary antibodies (goat or mouse anti-rabbit IgG) at room temperature for 1 h. Relative expression of proteins was analyzed using Odyssey software.

### Observation of Tumor Growth in vivo

When the volume of the tumor reached 90–100 mm^3^, HMME (10 mg/kg) was injected into the caudal vein (PDT group) of mice. The tumor site was irradiated with a 630-nm laser using a light power intensity of 120 J/cm^2^ after 4 h and 15 min was selected as the laser irradiation time. The control group was not administered PS or irradiation. The efficacy of treatment was evaluated on the basis of the sizes and weights of tumors once every 2–4 days for 16 days and compared with the control group. Tumor volume (V) was calculated using the formula: V = (a×b^2^)/2, where a is the longest diameter and b is the shortest diameter.

### HE Staining and IHC

Tumors from 20 mice in the PDT group and control group were cut and fixed with 10% formalin overnight. Specimens were embedded in paraffin and cut into 4-µm-thick sections before undergoing light microscopic examination. HE staining was used to observe the histological changes of the tumor; expression of caspase-1, caspase-3, caspase-6, caspase-9, and PARP proteins were evaluated by IHC *in vivo*.

### Statistical Analysis

Data were calculated and analyzed using SPSS13.0 software and were obtained from 3 independent experiments. Statistical analysis was conducted using Student’s *t* -test and one-way analysis of variance. *P*-values less than 0.05 were considered statistically significant.
